# Protective Potential of β-Hydroxybutyrate against Glucose-Deprivation-Induced Neurotoxicity Involving the Modulation of Autophagic Flux and the Monomeric Aβ Level in Neuro-2a Cells

**DOI:** 10.3390/biomedicines11030698

**Published:** 2023-02-24

**Authors:** Yi-Fen Chiang, Ngan Thi Kim Nguyen, Shih-Min Hsia, Hsin-Yuan Chen, Shyh-Hsiang Lin, Ching-I Lin

**Affiliations:** 1 School of Nutrition and Health Sciences, College of Nutrition, Taipei Medical University, Taipei 11031, Taiwan; 2 Programs of Nutrition Science, School of Life Science, National Taiwan Normal University, Taipei 10610, Taiwan; 3 School of Food Safety, College of Nutrition, Taipei Medical University, Taipei 11031, Taiwan; 4 Graduate Institute of Metabolism and Obesity Sciences, College of Nutrition, Taipei Medical University, Taipei 11031, Taiwan; 5 Nutrition Research Center, Taipei Medical University Hospital, Taipei 11031, Taiwan; 6 Department of Nutrition and Health Sciences, Kainan University, Taoyuan 338, Taiwan

**Keywords:** glucose deprivation, β-hydroxybutyrate, monomeric beta-amyloid peptides, autophagic flux, neurodegenerative diseases, Alzheimer’s disease

## Abstract

Hypoglycemia has been known as a potential contributory factor to neurodegenerative diseases, such as Alzheimer’s disease. There may be shared pathogenic mechanisms underlying both conditions, and the ketone body, β-hydroxybutyrate (BHB), as an alternative substrate for glucose may exert neuroprotection against hypoglycemia-induced injury. To investigate this, Neuro-2a cells were subjected to a 24 h period of glucose deprivation with or without the presence of BHB. Cell viability, reactive oxygen species (ROS) production, apoptosis, autophagy, and adenosine triphosphate (ATP) and beta-amyloid peptide (Aβ) levels were evaluated. The results show that Neuro-2a cells deprived of glucose displayed a significant loss of cell survival with a corresponding decrease in ATP levels, suggesting that glucose deprivation was neurotoxic. This effect was likely attributed to the diverse mechanisms including raised ROS, defective autophagic flux and reduced basal Aβ levels (particularly monomeric Aβ). The presence of BHB could partially protect against the loss of cell survival induced by glucose deprivation. The mechanisms underlying the neuroprotective actions of BHB might be mediated, at least in part, through restoring ATP, and modulating ROS production, autophagy flux efficacy and the monomeric Aβ level. Results imply that a possible link between the basal monomeric Aβ and glucose deprivation neurotoxicity, and treatments designed for the prevention of energy impairment, such as BHB, may be beneficial for rescuing surviving cells in relation to neurodegeneration.

## 1. Introduction

The neurons in the hippocampus and cerebral cortex are responsible for the function of learning and memory, and are the most vulnerable to damage from hypoglycemia [1]. Hypoglycemia is commonly associated with the treatments of diabetes, but contributes to increased risks of neurodegenerative diseases, i.e., dementia and cognitive impairment, in diabetic patients [2]. Intriguingly, a growing body of evidence suggests that reduced glucose metabolism is a pivotal pathogenic component for Alzheimer’s disease (AD) which is the most common cause of dementia [3]. Epidemiological evidence reveals that elderly diabetic patients who experience severe hypoglycemia are at greater risk of developing dementia and AD [4]. If hypoglycemia is not corrected with glucose immediately, brain glucose deprivation can occur, leading to severe brain injury. Animal studies have demonstrated that insulin-induced hypoglycemia causes significant neuronal cell death and cognitive impairment in rats, and in an in vitro study, the exposure of cortical neurons to glucose deprivation also manifests neuronal cell death [5]. Collectively, glucose deprivation could contribute to the pathogenesis of neurodegenerative diseases such as AD.

In response to nutrient starvation or energy deprivation, an adaptive mechanism at the cellular level, namely macroautophagy (hereafter referred to as autophagy), can be triggered to promote cell survival via the maintenance of cellular energy homeostasis [6]. Under normal conditions, autophagy is a self-degradative pathway for eliminating cytoplasmic constituents including abnormal protein aggregates, defective proteins and organelles, which is pivotal to maintaining cellular homeostasis [7,8]. Autophagy in neurons appears to play a critical role in protecting against aging and relevant neurodegenerative diseases [9,10]. For instance, a dysfunctional autophagy pathway has been reported to be involved in hypoglycemia-induced neuronal cell death [11] as well as neurodegenerative proteinopathies (e.g., Aβ accumulation) [10]. In the healthy brain, a balance between Aβ production and its removal is maintained by the competent autophagy machinery, indicating that Aβ accumulation is likely prevented [10]. The accumulation of pathogenic Aβ in AD may be the consequence of dysregulated autophagy [12,13,14]. As mentioned above, impaired glucose metabolism and Aβ accumulation may be involved in the pathogenesis of AD; however, whether both events shared a common mechanism in relation to autophagy remains unclear.

Ketone bodies, which include acetoacetate, β-hydroxybutyrate (BHB) and acetone, can act as alternative energy substrates in the brain during the conduction of a limited glucose supply, e.g., prolonged fasting, starvation and hypoglycemia, in order to prevent the loss of brain function. Several animal studies have reported the protective effects of BHB against hypoglycemia-induced neuronal cell death [15,16]. Likewise, in vitro studies associated with energy failure such as glucose deprivation and glycolysis inhibition have shown the potential of BHB to prevent neuronal cell death [16,17]. The mechanisms underlying the potential neuroprotective actions of BHB may be attributable to its antioxidant and autophagy stimulation properties [17,18,19]. Interestingly, a previous in vitro study has shown that BHB exerts putative neuroprotection against Aβ-induced neuronal cell death [20]. However, whether the mechanism by which BHB attenuates Aβ neurotoxicity is related to autophagy remains elusive. Although there are studies investigating the beneficial effects of BHB under the condition of glucose deprivation in vitro, none of them has explored the association between Aβ production and the neurotoxicity of glucose deprivation or the neuroprotective potential of BHB. Given the possible involvement of hypoglycemia in the pathogenesis of AD, we made an attempt to ascertain whether metabolic disturbances in glucose deprivation showed a possible link between neuronal cell death and subsequent changes in Aβ levels, and what role BHB played in this event.

Therefore, in the present study, we hypothesized that the ketone body, BHB, could prevent neuronal cell death under a condition of glucose deprivation through restoring ATP, stimulating autophagy, and modulating the levels of Aβ and ROS. To test this, the mouse Neuro-2a neuroblastoma cell line was exposed to a 24 h period of glucose deprivation condition with or without the presence of BHB, and cell survival, autophagy function, ROS production and Aβ and ATP levels were determined.

## 2. Materials and Methods

### 2.1. Cell Culture β-Hydroxybutyrate Treatment and Bafilomycin A1 Pre-Treatment

The mouse Neuro-2a neuroblastoma cell lines were obtained from the Bioresource Collection and Research Center (BCRC, No. 60026, Hsinchu, Taiwan). This cell line was chosen because it is of neuronal origin [21] and has been used in AD research regarding Aβ cytotoxicity [22,23]. Briefly, the Neuro-2a cells were then cultured in Dulbecco’s modified Eagle Medium (DMEM, Gibco, Grand Island, NY, USA) containing 25 mM glucose supplemented with 10% fetal bovine serum (FBS, CORNING, Manassas, VA, USA) and 100X penicillin streptomycin solution (CORNING, Christiansburg, VA, USA) at 37 °C in a humidified 5% CO_2_ incubator. The Neuro-2a cells cultured in this glucose-containing basal condition served as a control group, which is further referred to as the “C” condition. For the experimental condition of glucose deprivation, the Neuro-2a cells were cultured in non-glucose DMEM (Gibco, Grand Island, NY, USA) with the same environment, which is further referred to as the “N” condition. For β-hydroxybutyrate (BHB, Sigma-Aldrich, St. Louis, MO, USA) treatment, 10 mM BHB was added simultaneously with the non-glucose DMEM and was then used for the Neuro-2a cell cultures. Thus, the Neuro-2a cells were cultured in non-glucose DMEM with the presence of 10 mM BHB, which is further referred to as the “B” condition. To monitor the effects of glucose deprivation with or without the presence of BHB on autophagy, an autophagosome–lysosome fusion inhibitor, bafilomycin A1 (BAF, Cayman Chemical Company, Ann Arbor, MI, USA) was added to the glucose-containing medium with a concentration of 100 nM. The Neuro-2a cells were then cultured in this medium for 4 h at 37 °C in a humidified 5% CO_2_ incubator. After pre-incubation with BAF, the medium was removed and a fresh glucose-containing medium or non-glucose medium with or without the presence of BHB was added, followed by incubation for another 24 h. With the BAF pre-treatment, therefore, the Neuro-2a cells cultured in glucose-containing DMEM, and non-glucose DMEM with or without the presence of BHB are further referred to as the “BAF”, “N + BAF” and “B + BAF” conditions, respectively.

### 2.2. Cell Viability Assessment

Cell viability was evaluated by the use of 3-(4,5-dimethyl thiazol)-2,5-diphenyltetrazolium bromide (MTT) assays. Briefly, 3 × 10^3^ cells were seeded into a 96-well cell culture plate. The next day, cells were cultured in the C, N or B conditions for 8, 24 and 48 h. Cell survival was further monitored at respective time points by adding 100 μL of the 0.1 mg/mL MTT, and the cells were kept in a 37 °C CO_2_ incubator for 3 h. After dissolving formazan crystals in dimethyl sulfoxide (DMSO) solution, cell survival was judged based on the absorbance of this colored solution, which was measured using an ELISA reader at the wavelengths of 570 and 630 nm. In addition, MTT assays were also employed to evaluate the role of glucose deprivation in autophagy-induced cytotoxicity. For this purpose, Neuro-2a cells were pre-treated with BAF for 4 h and then the culture medium was replaced with either a glucose-containing basal medium or non-glucose medium with or without the presence of 10 mM β-hydroxybutyrate followed by incubation for another 24 h. After treatments, the Neuro-2a cells were subjected to MTT assays following the aforementioned protocol.

### 2.3. Cell Counting Trypan Blue Stain Assay

Further, trypan blue stain assay was conducted for the determination of a viable cell count in order to confirm the cytotoxic effects of experimental conditions. Neuro-2a cells were cultured in a 6-well plate (2 × 10^5^ cells/well) under C, N or B conditions for 8, 24 or 48 h. After incubation, cells were detached by trypsinization. The cell pellet was collected and re-suspended in PBS solution followed by staining with trypan blue (CORNING, Christiansburg, VA, USA). The viable cells, which did not take up the stain, were counted with a hemocytometer under a microscope (Olympus, Tokyo, Japan).

### 2.4. Assessment of Intracellular ROS Generation

Quantification of oxidative stress in Neuro-2a cells exposed to the C, N or B conditions was carried out by measuring total ROS using 2′,7′-dichlorofluorescin diacetate (DCFDA, Cayman, Ann Arbor, MI, USA) staining. The non-fluorescent DCFDA can serve as an indicator for ROS, because it can be rapidly oxidized to form fluorescent 2′,7′-dichlorofluorescein (DCF) in the cells with the presence of ROS. In brief, after Neuro-2a cells were cultured in the C, N and B conditions for 24 h, cells were incubated with 25 μM DCFDA for 30 min to detect total ROS by quantifying the fluorescence intensity of DCF. Next, microscopy was used to produce the fluorescence image, and the intensity of fluorescent DCF in a single cell was quantified using ImageJ software (Version 1.52t, NIH, Bethesda, MD, USA).

### 2.5. Propidium Iodide Staining

After the Neuro-2a cells were cultured in the C, N and B conditions for 24 h, the cells were stained with PI (1 μg/mL, dissolved with sterile ddH2O, Sigma-Aldrich, St. Louis, MO, USA) solution for 1 h. Fluorescence was monitored at 200× magnification using microscopy (Olympus, Tokyo, Japan). To compare the fluorescence intensity in Neuro-2a cells among the groups, Image J software (Version 1.52t, NIH, Bethesda, MD, USA) was used to quantify the fluorescence intensity in a single cell. In brief, after 4–6 images (each image representing one biological replicate) of each group were taken at random locations, three different cells representing technical replicates in each image were randomly selected to determine the level of fluorescence with the ImageJ software.

### 2.6. Assessment of ATP Concentrations and ATP/ADP Ratios

After Neuro-2a cells were cultured in the C, N and B conditions for 24 h, cells were further subjected to the analysis of ATP concentration and ATP/ADP ratio by ADP/ATP Ratio Bioluminescent Assay Kit (BioVision, Cambridge, UK) with a luminometer. The assays were performed according to the manufacturer’s instructions. Briefly, the background luminescence value was defined as data A. After treating the Neuro-2a cells in the control or experimental conditions, the culture medium was removed and the cells were treated with a nucleotide-releasing buffer for 5 minutes at room temperature, and the luminescence values were measured and considered data B. Then, this value was read again as data C for determining the ADP levels. Finally, the luminescence values were determined again as data D followed by the addition of an ADP-converting enzyme to activate the reaction. ATP concentration and the ATP/ADP ratio were calculated according to the following formulas: ATP= data B − data A and ATP/ADP ratio= (data B − data A)/(data D − data C), respectively.

### 2.7. Western Blot Analysis

After the Neuro-2a cells were cultured in the C, N, B, BAF, N + BAF or B + BAF conditions for 24 h, the cell lysates were prepared in an ice-cold lysis buffer (50 mmol/L Tris (pH 8.0), 100 mmol/L sodium chloride (NaCl), 0.1% sodium dodecyl sulfate (SDS), 1% NP-40 and 0.5 mM ethylene diamine tetra acetic acid (EDTA) containing the protease (Roche, Basel, Switzerland). The proteins (30 μg) were boiled for 5 min, separated using 10% or 15% SDS-polyacrylamide gel electrophoresis, and then transferred onto Immobilon-P polyvinylidene fluoride membranes (0.22 µm) for 150–180 min at 280 mA and 250 V. Then, the membranes were washed three times in tris-buffered saline (TBS) plus Tween 20 (TBST) buffer for 10 min each, blocked with blocking buffer (5% BSA) for 1 h at room temperature and incubated overnight with primary antibodies, i.e., Aβ _1–42_ (1:1000) (Millipore, Rehovot, Israel), PARP (1:000) (Cell signaling, Danvers, MA, USA), p62 (1:1000) (Cell signaling, Danvers, MA, USA), LC3B (1:1000) (Cell signaling, Danvers, MA, USA), cathepsin B (1:1000) (Cell signaling, Danvers, MA, USA) and glyceraldehyde-3-phosphate dehydrogenase (GAPDH) (1:10,000) (Proteintech, Rehovot, Israel) at 4 °C. The next day, the membranes were washed three times in the TBST (tris-buffered saline with Tween 20) buffer for 10 min each, incubated for 1 h in the blocking buffer with anti-rabbit/mouse immunoglobulin G (IgG) coupled to alkaline phosphatase (1:10,000), and washed three times in the TBST buffer for 10 min each. Then, the bands were detected using enhanced chemiluminescence (ECL). The chemiluminescent signals were detected by the e-BLOT Touch Imager (e-BLOT, Shanghai, China). The values shown were quantified, normalized to the internal control GAPDH, and then densitometry estimation was performed using the ImageJ software (NIH, Bethesda, MD, USA). Original, uncropped images of Western blots are presented in Supplementary Figure S1.

### 2.8. Intracellular Aβ ELISA

After the Neuro-2a cells were cultured in the C, N or B conditions for 24 h, cells were collected with pre-cooling PBS and the cell lysates were prepared for intracellular Aβ determination. The levels of cellular Aβ were quantified by using the mouse Aβ ELISA kit (Novus Biologicals, Littleton, CO, USA) according to the manufacturer’s instructions with the determination of optical density at 450 nm by an ELISA plate reader.

### 2.9. Immunofluorescence Analysis

Co-localization of p62 and LC3B or cathepsin B was analyzed for monitoring the autophagic flux. After Neuro-2a cells were cultured in the C, N, or B conditions for 24 h, cells grown on coverslips were washed in PBS and fixed using 4% paraformaldehyde for 10 min at room temperature followed by PBS wash. The coverslips were then incubated in the permeabilization solution containing 0.5 % Triton X-100 in PBS for 10 min at room temperature. After washing three times with PBS, the coverslips were blocked in a blocking solution containing 5% bovine serum albumin (BSA) in TBST for 30 min at room temperature. The coverslips were incubated with the primary antibodies, anti-p62 (1:1000) anti-LC3B (1:1000) and anti-cathepsin B (1:200, cell signaling) at appropriate dilutions overnight at 4 °C. After washing with PBS, the coverslips were incubated with the respective secondary antibodies, which were either Alexa Fluor 546-goat anti-mouse or Alexa Fluor 488-anti-rabbit IgG antibodies (1:200) (Life Technologies, Gaithersburg, MD, USA) for 1 h at room temperature. The nuclear counterstaining was performed with 4′,6-diamidino-2-phenylindole (DAPI) (Thermo Fisher Scientific, Waltham, MA, USA). The results were visualized under a fluorescence microscope (Olympus, Tokyo, Japan).

### 2.10. Statistical Analysis

All quantitative results are expressed as mean ± standard error of the mean (SEM), and were analyzed using the Prism version 6.0 software (GraphPad, San Diego, CA, USA). The Shapiro–Wilk test was performed to test the normality of the data. Because all datasets passed the Shapiro–Wilk normality tests with *p*-values greater than 0.05, the differences between the mean values of the three groups were then determined using one-way analysis of variance (ANOVA) followed by multiple comparisons with Tukey’s HSD post hoc test. Significance was accepted at *p* < 0.05.

## 3. Results

### 3.1. β-Hydroxybutyrate Confers Partial Neuroprotection against Glucose-Deprivation-Induced Neuro-2a Cell Death

**Figure 1:**
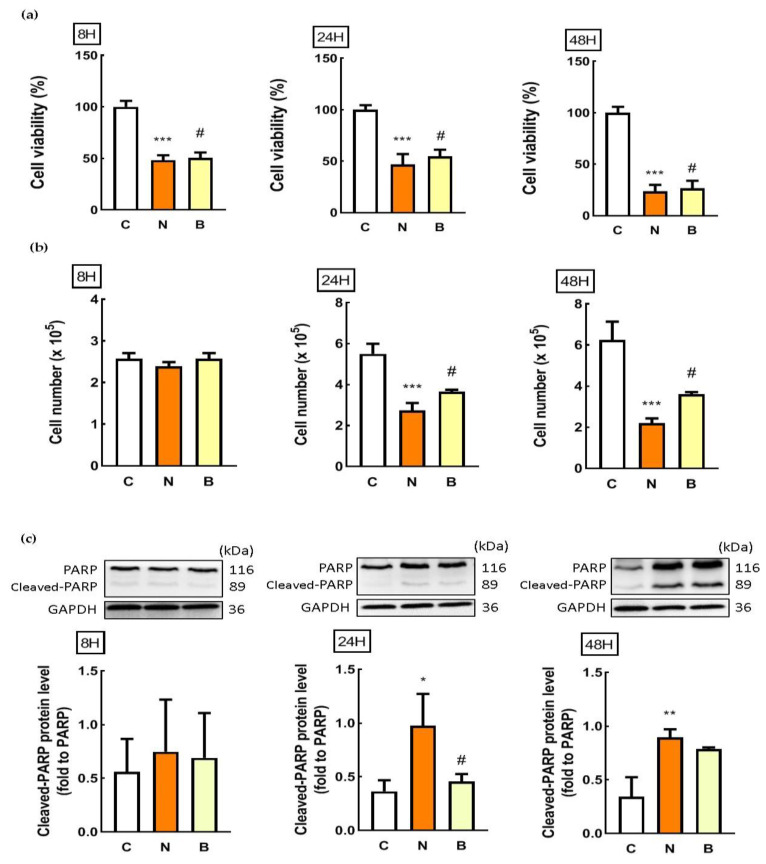
Effect of β-hydroxybuterate on glucose-deprivation-induced Neuro-2a cell death. Neuro-2a cells were cultured in glucose-containing basal conditions (C group), or in the condition of glucose deprivation with (B group) or without the presence of 10 mM β-hydroxybutyrate (N group) for 8, 24, and 48 h. Cell viability and counting was determined by (**a**) MTT and (**b**) trypan blue assays, respectively. Data are means ± SEM of 4–6 independent experiments, each with six replicates. Whole cell lysates from all conditions were subjected to Western blot analysis for the detection of (**c**) cleaved-PARP protein expression. Representative Western blot images are shown. GAPDH was used as the loading control. Data are means ± SEM of 4–6 independent experiments, and each experiment was performed in simplicate. Statistical analysis was performed with one-way ANOVA followed by a Tukey’s HSD post hoc test. *, *p* < 0.05; **, *p* < 0.01; ***, *p* < 0.001 compared with the control group. #, *p* < 0.05 compared with the N group.

Cell viability and trypan blue assays were used to evaluate cytotoxicity. In parallel, Western blot analysis was also performed for detecting poly ADP-ribose polymerase (PARP) cleavage. This cleavage has been recognized as an indication of cell death [5]. As shown in Figure 1a, glucose-deprived Neuro-2a cells, regardless of the presence of BHB, exhibited a marked decrease in cell viability in a time-dependent manner. Relative to control exposure (C group), cell viability in response to glucose deprivation decreased significantly at all times analyzed, with the 48 h exposure demonstrating the greatest decrease (*p* < 0.001, Figure 1a). When compared to the glucose-deprived Neuro-2a cells in the absence of BHB (N group), the presence of BHB appeared to significantly reverse the glucose-deprivation-induced decrease in Neuro-2a cell viability at all times analyzed (B group, *p* < 0.05, Figure 1a). Glucose deprivation with the presence of BHB for 24 h (B group) displayed maximal suppression of neuronal cell death (*p* < 0.05, Figure 1a). Similar results were observed when cell survival was monitored by the trypan blue assay. The number of viable cells was not altered by glucose deprivation exposure for 8 h, but was significantly decreased by glucose deprivation exposure for 24 h and 48 h, regardless of the presence of BHB (*p* < 0.001, Figure 1b). Co-incubation with BHB for 24 h and 48 h significantly attenuated the decreased number of glucose-deprived Neuro-2a cells (*p* < 0.05, Figure 1b). Likewise, as shown in Figure 1c, the cleaved PARP protein expression was not altered after glucose deprivation exposure for 8 h, but was significantly increased after glucose deprivation exposure for 24 h and 48 h, indicating that glucose deprivation could induce Neuro-2a cell death in a time-dependent manner. The glucose deprivation-mediated increase in the cleaved PARP protein expression of Neuro-2a cells was profoundly decreased by co-incubation with BHB for 24 h (Figure 1c). These results suggest that the glucose deprivation was likely cytotoxic for Neuro-2a cells leading to cell death in a time-dependent manner. Co-incubation with BHB during glucose deprivation appeared to partially alleviate these glucose-deprivation-induced cytotoxic effects in Neuro-2a cells. Moreover, according to Figure 1, due to the potent induction of half Neuro-2a cell loss by glucose deprivation exposure for 24 h, and the maximal suppression of glucose-deprivation-induced cytotoxicity by co-incubation with BHB for 24 h, a 24 h period of glucose deprivation exposure and resulting deleterious effects were chosen for further experiments.

### 3.2. β-Hydroxybutyrate Exerts Partial Neuroprotection against Glucose-Deprivation-Induced Neuronal Cell Death through the Normalization of Intracellular ROS

**Figure 2:**
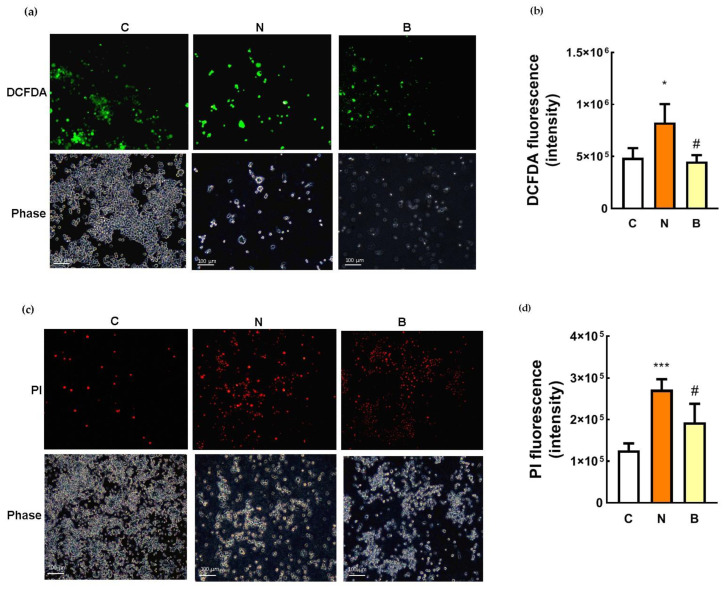
Effect of β-hydroxybutyrate on glucose-deprivation-induced oxidative stress and neuronal cell death. Neuro-2a cells were cultured in the glucose-containing basal condition (C group), or in the condition of glucose deprivation with (B group) or without the presence of 10 mM β-hydroxybutyrate (N group) for 24 h. After exposure to the different conditions, intracellular ROS generation was determined by DCFDA staining, and neuronal cell death was determined by propidium iodide (PI) staining. (**a**) Representative images of DCFDA staining showing the generation of ROS within Neuro-2a cells, which is visualized in green; phase contrast images are also shown. (**b**) Quantification of DCFDA fluorescence. (**c**) Representative images of PI staining showing the dead cells which are visualized in red; phase contrast images are also shown. (**d**) Quantification of PI fluorescence. Scale bars, 100 μm. Data are means ± SEM of 4–6 independent experiments, and each experiment was performed in simplicate. Statistical analysis was performed with one-way ANOVA followed by a Tukey’s HSD post hoc test. *, *p* < 0.05; ***, *p* < 0.001 compared with the control group. #, *p* < 0.05 compared with the N group.

It has been reported that glucose-deprivation-induced neurotoxic response is the result of oxidative stress in PC12 cells [24]. We also confirmed this observation in glucose-deprived Neuro-2a cells without BHB treatment (N group), the green fluorescence (DCFDA) of which was significantly enhanced in comparison with that of the control cells (C group) as can be viewed in the representative images (Figure 2a) and quantitative data (*p* < 0.05, Figure 2b). A marked increase in ROS production was observed within glucose-deprived Neuro-2a cells without BHB treatment, but this effect was able to be completely abolished by the presence of BHB (B group, *p* < 0.05, Figure 2b). BHB treatment showed a significant reduction in ROS generation within glucose-deprived cells with low green fluorescent intensity, which was similar to that observed in the control cells (Figure 2b), suggesting the inhibitory effect of BHB on intracellular ROS production. When overproduction of ROS was induced in the glucose-deprived Neuro-2a cells, an increase in neuronal cell death determined by PI staining was simultaneously observed (Figure 2c,d). As shown in the representative images (Figure 2c) and quantitative data (Figure 2d), the PI signal was significantly stronger in the glucose-deprived Neuro-2a cells, regardless of the presence of BHB, than in the control cells, indicating that increased neuronal cell death could be attributed to the excessive generation of ROS induced by glucose deprivation. However, this effect was partially reversed by the presence of BHB (Figure 2c,d), suggesting that BHB could act through scavenging intracellular ROS, at least in part, to protect against Neuro-2a cell death induced by glucose deprivation.

### 3.3. The Neuroprotective Role of β-Hydroxybutyrate Correlates with Cellular Energy Status

**Figure 3:**
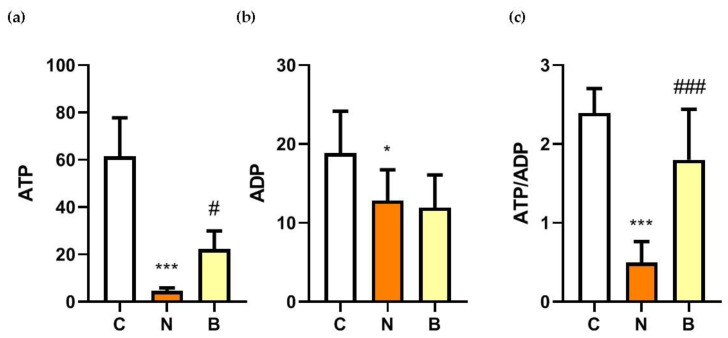
Effect of β-hydroxybutyrate on glucose-deprivation-induced changes in ATP and ADP concentrations, and ADP/ATP ratios. Neuro-2a cells were cultured in the glucose-containing basal condition (C group), or in the condition of glucose deprivation with (B group) or without the presence of 10 mM β-hydroxybutyrate (N group) for 24 h. Changes in (**a**) adenosine triphosphate (ATP) and (**b**) adenosine diphosphate (ADP) concentrations, and (**c**) the ratios of ATP to ADP are shown. Data are means ± SEM of 4–6 independent experiments, each with three replicates. Statistical analysis was performed with one-way ANOVA followed by a Tukey’s HSD post hoc test. *, *p* < 0.05; ***, *p* < 0.001 compared with the control group. #, *p* < 0.05; ###, *p* < 0.001 compared with the N group.

To investigate whether the neuroprotective action of BHB correlated with changes in cellular energy status, adenosine triphosphate (ATP) concentrations and ATP/adenosine diphosphate (ADP) ratios were determined after glucose deprivation exposure with or without the presence of BHB for 24 h. As shown in Figure 3, ATP and ADP concentrations as well as the ratio of the two were significantly reduced in the glucose-deprived Neuro-2a cells without the presence of BHB (N group) compared to those of the control cells (*p* < 0.05, Figure 3). As expected, these results show a reduced cellular energy status due to glucose deprivation. During glucose deprivation, ATP concentration and the ratio of ATP to ADP were significantly higher in the BHB-treated Neuro-2a cells (B group) than in the untreated cells (N group) (*p* < 0.05, Figure 3a,c), indicating that glucose-deprivation-induced ATP reduction could be restored, at least in part, by BHB. These data suggest that a metabolic action in relation to the preservation of ATP could partially contribute to the neuroprotective effect of BHB against glucose-deprivation-induced neuronal cell death.

### 3.4. β-Hydroxybutyrate Partially Improves Glucose-Deprivation-Induced Impaired Autophagy Flux

**Figure 4:**
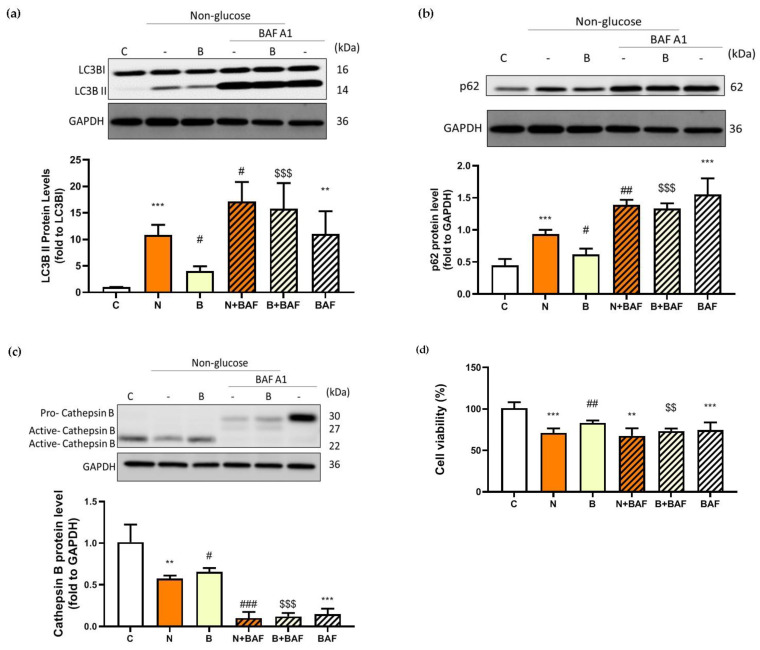

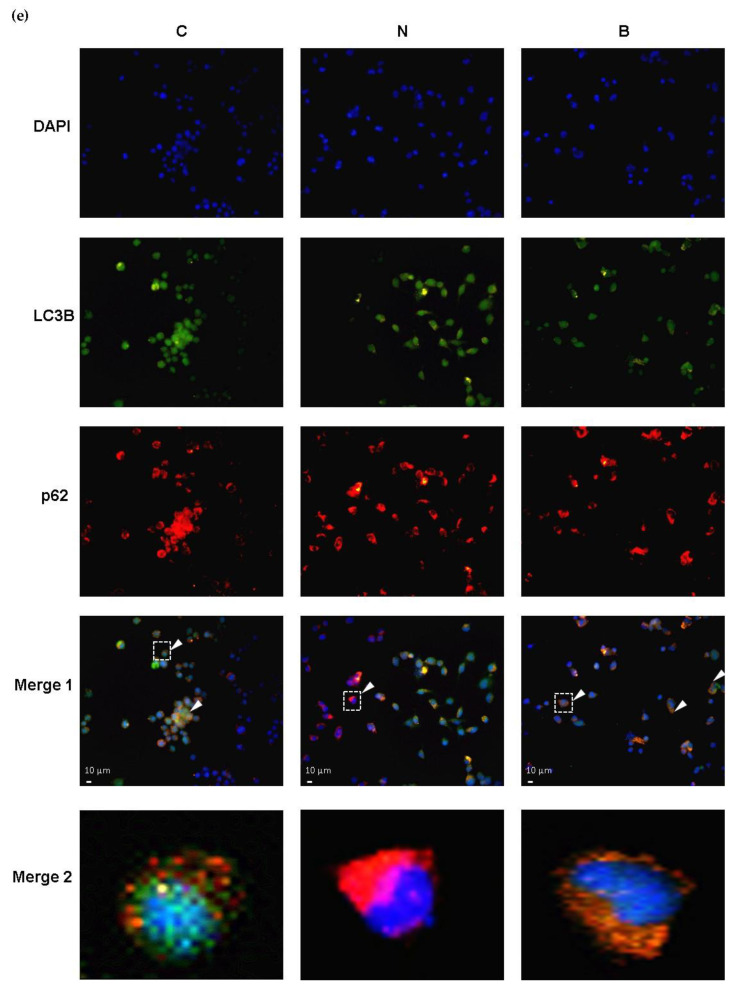

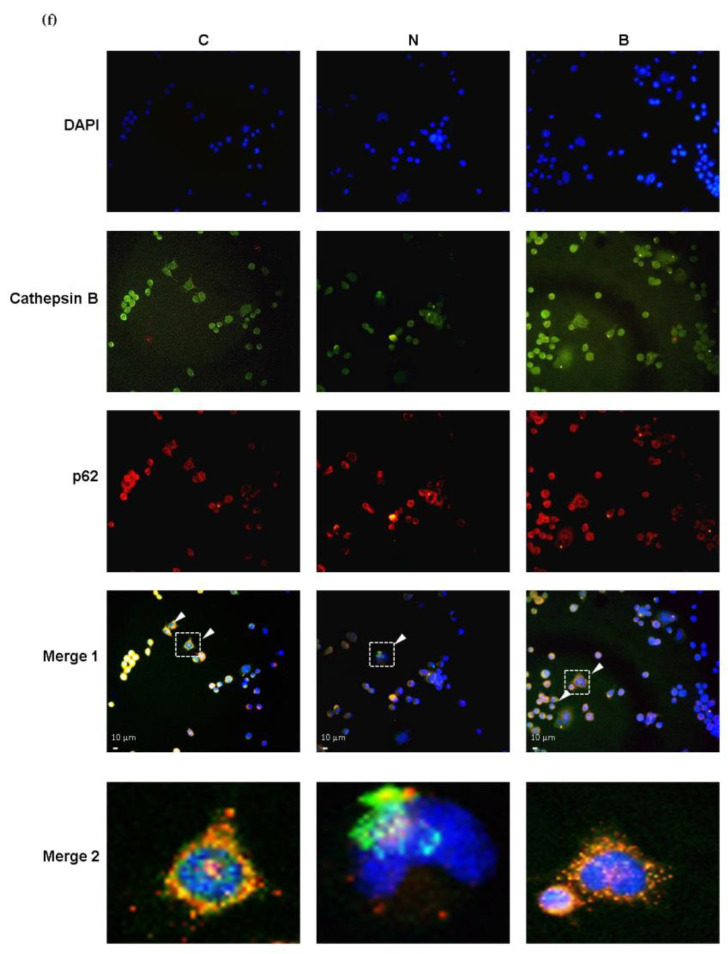
Effect of β-hydroxybutyrate on glucose-deprivation-induced changes in autophagy flux. Neuro-2a cells were cultured in the glucose-containing basal condition (C group), or in the condition of glucose deprivation with (B group) or without the presence of 10 mM β-hydroxybutyrate (N group) for 24 h. With the aim of inhibiting autophagy flux, Neuro-2-a cells were pre-treated with the autophagosome–lysosome fusion inhibitor, 100 nM bafilomycin A1 (BAF) for 4 h. After pre-treating with BAF, the culture medium was replaced with either the glucose-containing basal medium (BAF group) or the non-glucose medium with or without the presence of 10 mM β-hydroxybutyrate (B + BAF or N + BAF groups, respectively), and was incubated for 24 h. After whole cell lysates were prepared, Western blot analysis was performed to detect the expression of specific proteins including (**a**) the LC3B-II/LC3B-I ratio representing the conversion of LC3-I to LC3-II, (**b**) p62 and (**c**) cathepsin B. (**d**) Cell viability was determined by MTT assays. In the absence of BAF pre-treatment, co-localization of autophagy proteins was analyzed by immunofluorescence. Co-localization (merge fluorescence as indicated by arrowheads and a dashed line square in the merged image 1) of p62 (red fluorescence) and (**e**) LC3B (green fluorescence) or (**f**) cathepsin B (green fluorescence) is visualized within the Neuro-2a cells cultured in the C, N and B conditions. Co-localization of p62 and (**e**) LC3B or (**f**) cathepsin B for all groups is further shown in the merged image 2 by increasing magnification of the dashed line square area located in the merged image 1 of all groups. DAPI was used as a counterstain to visualize cell nuclei (blue fluorescence). The scale bar represents 10 μm. Representative Western blot images are shown. GAPDH was used as the loading control. Data are means ± SEM of 4–6 independent experiments, and each experiment was performed in simplicate. Statistical analysis was performed with one-way ANOVA followed by a Tukey’s HSD post hoc test. **, *p* < 0.01; ***, *p* < 0.001 compared with the control group. #, *p* < 0.05; ##, *p* < 0.01; ###, *p* < 0.001 compared with the N group. $$, *p* < 0.01; $$$, *p* < 0.001 compared with the B group.

To evaluate the autophagy efficacy, the expression of microtubule-associated protein 1 light chain-3B (LC3B-I/C3B-II), p62 and cathepsin B proteins were analyzed, all of which are recognized as autophagy markers at different steps of autophagy. During the induction of autophagy, autophagosome formation is a concomitant of the conversion of LC3B-I to LC3B-II, and therefore increased levels of LC3B-II may indicate accumulation of autophagosomes resulting from autophagy induction [10,25]. The p62 protein, also known as sequestosome1 (SQSTM1), acts as an autophagy adapter and substrate enabling the interaction with LC3B and thereby undergoing autolysosomal degradation [8,25], while cathepsin B is one of the abundant lysosomal proteases and is responsible for lysosomal degradation [26]. In this context, changes in the levels of both proteins may indicate the capacity of autophagic degradation or autophagic flux [25,27]. In the present study, autophagic flux was also determined by employing an autophagosome–lysosome fusion inhibitor, bafilomycin A1 (BAF) [28], which allowed us to confirm whether glucose-deprived cells displayed defective or component autophagy machinery. As shown in Figure 4a–c, in the absence of BAF and BHB treatments, significant increases in the LC3B-II/ LC3B-I ratio and expression of p62 protein accompanied by a significant reduction in expression of cathepsin B protein were observed in Neuro-2a cells under glucose deprivation (N group), compared to those of the control cells (C group, *p* < 0.01). These observations imply that either induction of autophagy or impaired autophagic flux might likely occur in Neuro-2a cells under glucose deprivation. Accumulation of autophagosomes accompanied by the suppression of autolysosomal degradation could be the result of impaired autophagic flux. It is worth noting that an induction of autophagy or upregulation of autophagic flux is generally characterized by an increased LC3B-I to LC3B-II conversion with a concomitant decrease in p62 expression [25]. To more accurately evaluate autophagy capacity, inhibition of autophagosome–lysosome fusion with BAF appeared to cause profound changes in all autophagy markers in glucose-deprived cells, regardless of the presence of BHB (Figure 4a–c). The LC3B-II/ LC3B-I ratio and p62 protein expression in the N + BAF group was statistically further increased from those in the N group (Figure 4a,b). Furthermore, the BAF inhibitor caused an even more marked reduction in cathepsin B expression for all groups (*p* < 0.001, Figure 4c). This finding further supports the assumption that the autophagosome accumulation was likely the consequence of a blockage of autophagosome–lysosome fusion by glucose deprivation. In other words, presumably, Neuro-2a cells exposed to a 24 h period of glucose deprivation could result in impaired autophagy flux. However, this adverse effect could be partially reversed by the presence of BHB as evidenced by the results showing that co-incubation with BHB during glucose deprivation significantly inhibited the increase in the LC3B-II/ LC3B-I ratio and p62 expression, and the reduction in cathepsin B expression (*p* < 0.05, Figure 4a–c). Additionally, the reduction in cell viability in the N group was notable, but was significantly decreased by the pre-treatment with BAF (N + BAF group, *p* < 0.01, Figure 4d). It seemed that BAF pre-treatment could augment the cytotoxic effect of glucose deprivation on Neuro-2a cells, suggesting a critical role of autophagosome–lysosome fusion in neuronal survival under glucose deprivation. These findings support a previous study showing that competent autophagy machinery is important for neuronal cell survival [29]. On the other hand, during a 24 h period of glucose deprivation with the presence of BHB, BAF pre-treatment had no additional effect on the loss of cell survival (B + BAF group, *p* > 0.05, Figure 4d) implying that energy is likely required for autophagy machinery to be completely fulfilled. The presence of BHB seemed to elicit a modulatory effect favoring improved autophagy flux, and such an effect could be potentially neuroprotective as BHB restored a loss of cell survival to a limited extent in parallel (Figure 4d). In agreement with these findings, the results of immunofluorescence showed that co-localization of p62 and LC3B (Figure 4e) or cathepsin B (Figure 4f) was also enhanced when Neuro-2a cells were cultured in the N condition. In addition, this effect was stronger after inhibition of autophagosome–lysosome fusion with BAF. The presence of BHB appeared to weaken this effect as shown in Figure 4e,f, where the signal of co-localization of p62 and LC3B or cathepsin B in the B group was weaker than in the N group. Taken together, these data suggest that glucose deprivation might lead to impaired autolysosomal degradation, thereby reducing autophagic flux efficacy, whereas the presence of BHB might partially suppress this effect.

### 3.5. The Neuroprotective Role of β-Hydroxybutyrate Correlates with Alteration in Intracellular Aβ Levels

**Figure 5:**
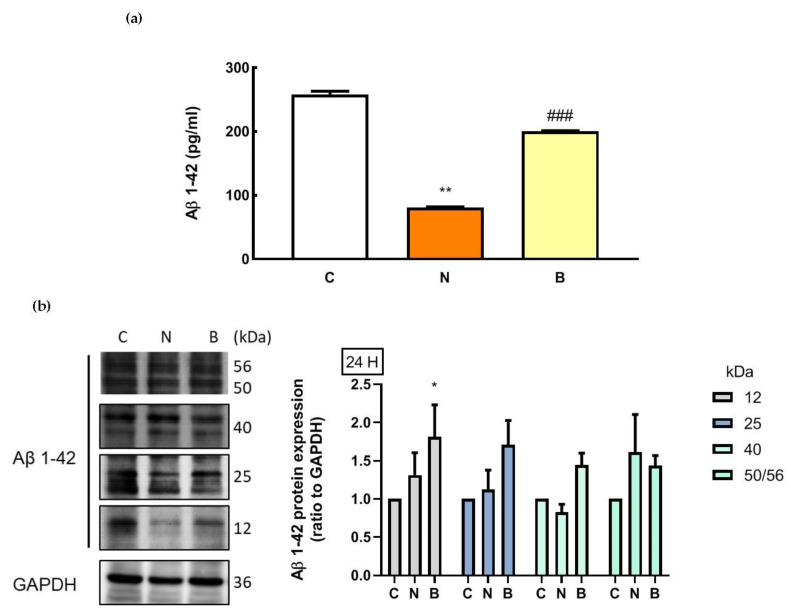
Effect of β-hydroxybutyrate on glucose-deprivation-induced changes in intracellular Aβ levels. Neuro-2a cells were cultured in the glucose-containing basal condition (C group), or in the condition of glucose deprivation with (B group) or without the presence of 10 mM β-hydroxybutyrate (N group) for 24 h. (**a**) Intracellular levels of monomeric Aβ were analyzed with an Aβ_1–42_-specific ELISA. (**b**) Changes in protein expression of oligomeric Aβ species were determined by Western blot analysis. Representative Western blot images are shown. GAPDH was used as a loading control. Data are means ± SEM of 4–6 independent experiments, and each experiment was performed in simplicate. Statistical analysis was performed with one-way ANOVA followed by a Tukey’s HSD post hoc test. *, *p* < 0.05; **, *p* < 0.01 compared with the control group. ###, *p* < 0.001 compared with the N group.

As mentioned above, Aβ accumulation in AD pathology is associated with defects in glucose metabolism and autophagy. However, under non-pathological conditions, Aβ may have crucial physiological roles [30]. On the basis of this concept, we investigated whether intracellular Aβ levels of Neuro-2a cells were affected by glucose deprivation with or without the presence of BHB. It is worth noting that amyloid plaques, the key pathological characteristic of AD, are formed and aggregated from different Aβ species including monomeric Aβ, oligomeric Aβ, protofibrils and fibrils [31]. The oligomeric Aβ is formed from the self-aggregation of the monomeric Aβ due to its hydrolytic properties [31]. Utilization of the enzyme-linked immunosorbent assay (ELISA) approach has allowed the quantification of monomeric Aβ species (molecular weight ~4 KDa) [31,32]. Changes in intracellular Aβ levels are shown in Figure 5a. Glucose deprivation itself resulted in a significant decrease in monomeric Aβ levels in the N group, compared to that of the control cells in the C group (*p* < 0.01, Figure 5a), and this decrease was significantly attenuated by co-incubation with BHB (*p* < 0.001). During glucose deprivation, BHB-treated Neuro-2a cells (B group) exhibited significantly higher levels of monomeric Aβ than untreated cells (N group, *p* < 0.001, Figure 5a). Furthermore, in order to detect intracellular oligomeric Aβ, Western blot analysis was performed in parallel. According to a previous study [33], oligomeric Aβ species can be shown as bands at 56 (dodecamer), 50, 40 (nonamer), 25 (hexamer) and 12 (trimer) kDa in a Western blot by the use of the Aβ antibody. As shown in Figure 5b, by using the same source of the Aβ antibody as Sandoval et al. [33] in their study, specific bands corresponding to 56, 50, 40, 25 and 12 kDa were observed within the intracellular fractions in all groups representing oligomeric Aβ species. However, no significant changes were found in the expression of the 56/50, 40 or 25 kDa bands among the groups (*p* > 0.05). The expression of the 12 kDa band was significantly higher in the B group than in the C and N groups (*p* < 0.05, Figure 5b). Nevertheless, the decreased levels of monomeric Aβ were likely associated with the loss of Neuro-2a cell survival after glucose deprivation exposure, and BHB might play a neuroprotective role, in part, in modulating the decreased monomeric Aβ level due to glucose deprivation.

## 4. Discussion

Previous evidence has shown that hypoglycemia could lead to neuronal cell death, which contributes to cognitive impairment and dementia including AD [2,3,5]. However, whether hypoglycemia plays a role in Aβ accumulation is unclear. Ketone bodies have been shown to elicit beneficial effects that pertain to autophagy regulation [17,19] and neuroprotection against neurodegenerative diseases [34], but whether it has the potential to modulate Aβ levels is unknown. In the present study, a 24 h period of glucose deprivation exposure was able to induce Neuro-2a cell death, which is in agreement with previous studies demonstrating that neurotoxicity is the consequence of glucose deprivation [35]. Furthermore, we assumed that its underlying mechanisms might involve inadequate ATP supply, excessive ROS production, autophagy dysregulation and aberrant Aβ levels in Neuro-2a cells under such a condition of stress. Our observation showing that glucose deprivation could cause an elevation in ROS production is in accordance with previous studies using different cells, such as primary neurons [24,36] and SH-SY5Y cell lines [35]. In addition, the reduced ATP levels and ATP/ADP ratios accompanied by increased neuronal cell death due to glucose deprivation in Neuro-2a cells suggests a possible association between insufficient ATP supply and neuronal cell death. It has been suggested that energy failure contributes to neuronal cell death, which has also been implicated in neurodegenerative diseases [37].

When cells are subjected to nutrient stress, cell survival is normally promoted by an activated autophagy, as described above; however, dysfunctional autophagy can cause autophagic cell death, in which the accumulation of autophagosomes is observed [35]. In the present study, the protein expression of LC3B-II relative to LC3B-I in Neuro-2a cells was upregulated during glucose deprivation, indicating the increased number of autophagosomes [25]. Although this speculation might be explained by the possibility of autophagy enhancement, an elevation in the number of autophagosomes is not necessarily caused by an enhancement of autophagy as it could also be caused by a blockage in lysosomal degradation occurring in the later stage of autophagy [25]. Therefore, distinguishing between autophagy enhancement and degradation inhibition by glucose deprivation was achieved by the assessment of autophagy flux. In the present study, a corresponding increase in p62 levels of Neuro-2a cells was observed, suggesting a likelihood of lysosomal degradation inhibition under glucose deprivation. This assumption was further confirmed by the addition of a BAF inhibitor, which pointed to a putative impairment of autophagic flux that might occur in the glucose-deprived Neuro-2a cells for 24 h. Our findings are in line with a previous study showing that glucose deprivation, albeit for 2 h, in cortical neurons resulted in autophagosome accumulation and an impaired autophagic flux accompanied by the increase in LC3B and p62 levels. The authors of this study attributed their results to glucose-deprivation-induced ATP depletion [38]. Furthermore, we assume that this defective autophagy resulting from glucose deprivation could also contribute to the Neuro-2a cell death. It has been reported that the autophagy machinery is intrinsically associated with neuronal cell survival in terms of cellular homeostasis [31].

In the present study, the detrimental effects of glucose deprivation on Neuro-2a cells were partially reversed by the co-incubation with BHB, suggesting the neuroprotective potential of BHB against glucose-deprivation-induced injury. Likewise, the neuroprotective effects of BHB have been demonstrated in various neuronal cell death models, which are associated with oxidative damage [16,18], glucose deprivation [16,17] and Aβ exposure [20]. Furthermore, we provide evidence that the putative neuroprotective mechanisms of BHB were likely attributed to the preservation of ATP, attenuation of ROS and partial restoration of autophagic flux and Aβ levels. The observed neuroprotective mechanisms of BHB in the present study are compatible with a previous study suggesting that BHB protecting against hypoglycemia-induced neuronal cell death is associated with the preservation of energy levels and the alleviation of ROS production [16]. Presumably, the metabolic and antioxidative actions are likely responsible for its neuroprotection. This inference is further supported by another study, in which BHB exerts its antioxidant effect by eliminating ROS production to protect against neuronal death in an in vitro model of hypoglycemia and by inhibiting lipid peroxidation to protect against hypoglycemia-induced oxidative damage in rats [18]. The authors in the aforementioned study indicated that BHB is a potential antioxidant due to its ability to directly scavenge hydroxyl radicals (^•^OH) [23].

In light of the fundamental importance of autophagy in promoting neuronal survivability associated with neurodegenerative diseases, it was our intention to elucidate the effect of BHB on autophagy and Aβ levels in Neuro-2a cells under glucose deprivation. In addition to the metabolic, antioxidant actions, BHB appeared to exert a putative autophagy-modulating effect in the present study. Consequently, a concomitant reduction in glucose-deprivation-induced Neuro-2a cell death was seen, which might be explained in part by the effect of BHB on modulating autophagy in favor of improved autophagic flux efficacy. This interpretation was supported by our observation that the presence of BHB during glucose deprivation resulted in significant attenuation of autophagosome accumulation and lysosomal degradation inhibition. Similarly, it has been revealed that BHB treatment is able to stimulate autophagic flux in cultured neurons deprived of glucose, thereby preventing neuronal cell death [17]. Furthermore, in a rodent model of severe hypoglycemia, it was demonstrated that neuronal cell survival is promoted by the treatment of BHB via improved autophagy flux efficacy resulting from the reduced accumulation of autophagosomes and enhanced degradation of p62 [19]. Collectively, this modulatory effect on autophagy flux could be the mechanism underlying the neuroprotective action of BHB on glucose-deprivation-injured Neuro-2a cells. Interestingly, it has been documented that autophagy itself is considered as an ATP-consuming process, in which ATP provides a driving force for reactions at different stages [39]. For instance, the late stage of autophagy is involved in the degradation of unwanted protein and damaged organelles within the lysosome, and this lysosome-mediated protein degradation has been proven to depend on the ATP supply [40]. Accordingly, the modulation of autophagic flux by BHB under glucose deprivation might be attributable to its metabolic role.

Autophagy is also important for metabolizing Aβ. The degradation of Aβ has been reported to occur in lysosomes as well as in other intracellular organelles [27,41]. Both in vitro and in vivo studies have shown that autophagy activation increases Aβ clearance [42,43]. Under physiological conditions, Aβ is a soluble component of cellular metabolism through the proteolytic cleavage of the neuron trans-membrane β-amyloid precursor protein by β- and γ-secretases, and there is equilibrium between Aβ production and its degradation [31,44]. It is possible that impaired autophagy could affect Aβ clearance. We then examined changes in the intracellular levels of Aβ. Intriguingly, we observed that the level of monomeric Aβ, but not oligomeric Aβ, was significantly reduced in Neuro-2a cells deprived of glucose, and this effect was partly reversed by the presence of BHB. Oligomeric Aβ has been considered to be neurotoxic [31,45], whilst monomeric Aβ has been reported to be neuroprotective [30]. In the present study, it is plausible that the loss of Neuro-2a cell survival after glucose deprivation exposure might be associated with the decreased levels of monomeric Aβ. Evidence from in vitro studies has revealed that monomeric Aβ can exert neuroprotective roles in inhibiting excitotoxic neuronal cell death [30] and apoptosis, and stimulating autophagy [14]. Importantly, a physiological role for monomeric Aβ in the glucose metabolism of the brain has been suggested, and this is crucial to the neuronal survival [46]. In the present study, the reversal of the decreased level of monomeric Aβ by the presence of BHB suggests a potential neuroprotective action of BHB for glucose-deprived Neuro-2a cells. Incidentally, there is evidence showing a link between hyperoxia-induced activation of autophagy and intra-lysosomal accumulation of Aβ in vitro [47]. However, owing to the fact that the total intracellular levels of Aβ were measured in the present study, whether intra-lysosomal Aβ degradation is also affected by glucose deprivation remains to be further investigated.

## 5. Conclusions

In conclusion, the results of the present study suggest that cell death induced by a 24 h period of glucose deprivation in Neuro-2a cells was apparent, and this neurotoxic effect was attributed to diverse mechanisms including insufficient ATP supply, raised ROS, defective autophagic flux and a reduced level of intracellular monomeric Aβ. Changes in monomeric Aβ levels underlining a possible role of monomeric Aβ in glucose-deprivation-induced neurotoxicity is noteworthy. These findings imply that elevating glucose availability may be beneficial, and that can be relevant to the prevention of neurodegenerative diseases [48]. On the other hand, the presence of BHB was able to partially suppress this neurotoxicity, suggesting that BHB is likely neuroprotective in Neuro-2a cells under glucose deprivation. We postulate that the energy metabolism in glucose-deprived Neuro-2a cells could be partly sustained by BHB treatment, which in turn, might render neuroprotection for Neuro-2a cells, at least in part, against glucose-deprivation-induced neurotoxicity. The putative neuroprotective effects of BHB on Neuro-2a cells deprived of glucose might be mediated through multiple mechanisms of action, i.e., mitigating ROS production and monomeric Aβ reduction, and improving autophagic flux efficacy. Not only metabolic, but also other neuroprotective-associated actions of BHB, may make it a promising candidate for the development of potential neuroprotective agents. Further studies are warranted to assess the potential of BHB as a neuroprotective agent in in vivo models of hypoglycemia in relation to neurodegeneration or neurodegenerative diseases.

## Data Availability

The data presented in this study are available on request from the corresponding author.

## Supplementary Materials

The following supporting information can be downloaded at: https://www.mdpi.com/article/10.3390/biomedicines11030698/s1, Figure S1: Original western blots.
