# Supplementary material for: Protective Potential of β-Hydroxybutyrate against Glucose-Deprivation-Induced Neurotoxicity Involving the Modulation of Autophagic Flux and the Monomeric Aβ Level in Neuro-2a Cells
Source: Biomedicines. 2023 Feb 24;11(3):698. doi: 10.3390/biomedicines11030698 (PMC10045359; doi:10.3390/biomedicines11030698)
Supplement: Supplementary file 1 [file biomedicines-11-00698-s001.zip › biomedicines-2225470-supplementary.pdf]

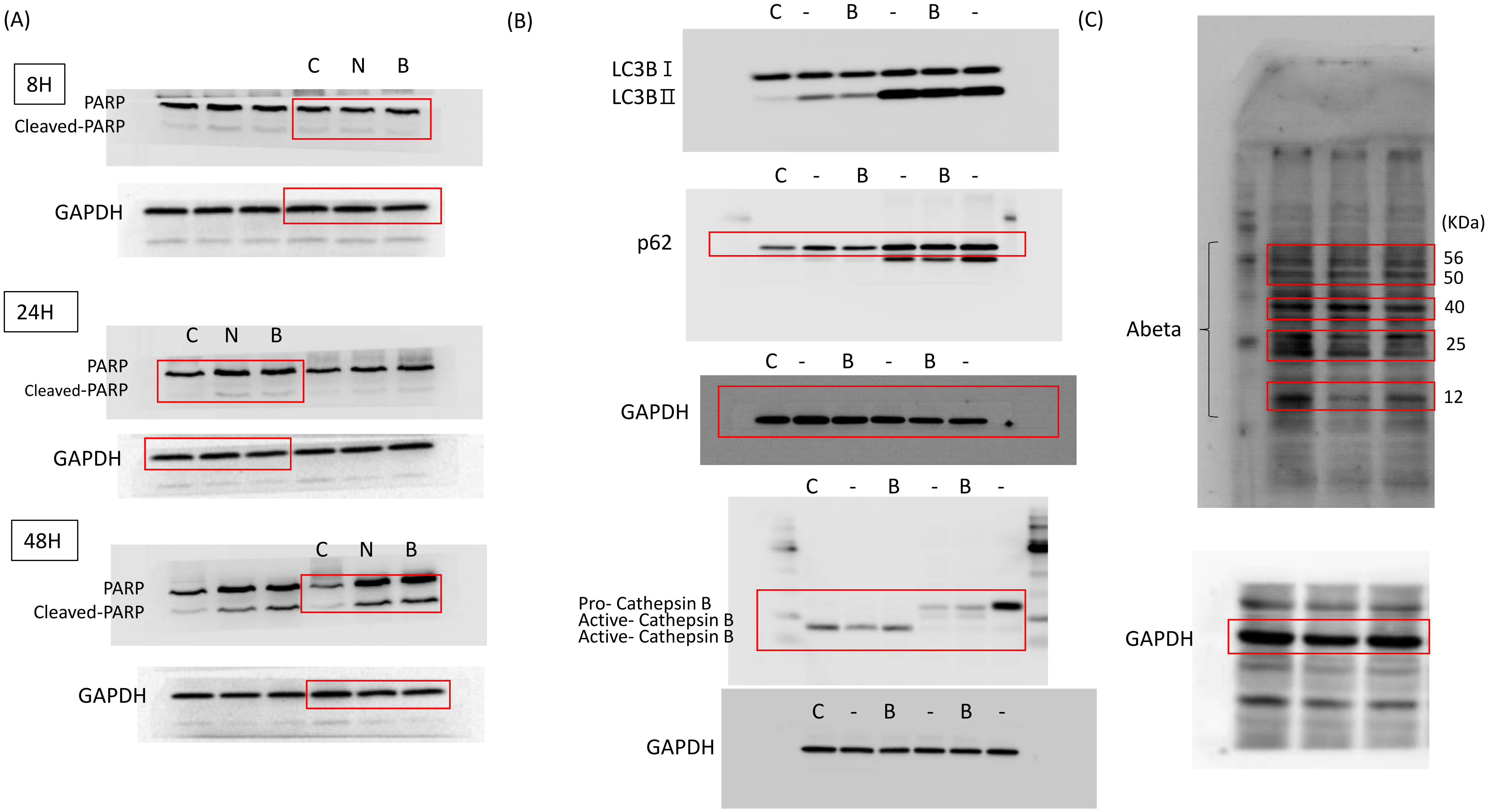

Figure S1 Original western blots. Original, uncropped images of Western blots related to (A) Figure 1c, (B) Figures 4a- 4c and (C) Figure 5b in the manuscript are shown and the areas taken for preparation of (A) Figure 1c, (B) Figure 4b and (C) Figure 5b are highlighted.
